# COVID-19 Test Us: A Case for Embedding Ethics and Regulatory Expertise

**DOI:** 10.1109/OJEMB.2022.3217431

**Published:** 2022-11-01

**Authors:** Holly A. Taylor, Janet F. Hale, Michael Centola, Allison Blodgett

**Affiliations:** Department of Bioethics, Clinical CenterNational Institutes of Health 2511 Bethesda MD 20892 USA; UMass Chan Medical School 12262 Worcester MA 01655 USA; Tan Chingfen Graduate School of Nursing 16117 Worcester MA 01655 USA; IRB Operations Worcester MA 01605 USA; Office of Research IntegrityUMass Lowell 14710 Lowell MA 01854 USA

**Keywords:** Covid-19, device testing, ethics, RADx

## Abstract

A key aspect of the National Institutes of Health (NIH) funded Rapid Acceleration of Diagnostics (RADx) Tech program was an active Clinical Studies Core including Committees with unique expertise to facilitate the development and implementation of studies to test novel diagnostic devices for Covid-19. The Ethics and Human Subjects Oversight Team (EHSO) was tasked to provide ethics and regulatory expertise to stakeholders in the RADx Tech effort. The EHSO developed a set of Ethical Principles to guide the overall effort and provided consultation on a wide range of ethical and regulatory concerns. Having access to a pool of experts with ethical and regulatory knowledge who met weekly to tackle issues of importance to the investigators was critical to the overall success of the project.

## I. Introduction

Think back to the start of the 2020 COVID-19 pandemic when there were no vaccines, therapeutics, or widely available diagnostics. There was an urgent need for rapid development and testing in these areas, but the social value of developing these products as quickly as possible had to be balanced with the need to minimize harm to the subjects willing to enroll.

The Rapid Acceleration of Diagnostics (RADx) Tech program was part of the overall National Institutes of Health (NIH) supported RADx initiative to speed innovation in the development, commercialization, and implementation of technologies for COVID-19 testing. University of Massachusetts Chan Medical School was the coordinating site for the RADx Tech COVID-19 Clinical Studies Core (CSC), which is publicly branded as “COVID-19 Test Us.” The effort required a multi-site, national effort in collaboration with academic, commercial, and community partners to test novel COVID-19 diagnostic technologies [1] [2] [3].^1^^1^Full list of publications at https://www.covid19testus.org/what-is-test-us/publications/

All RADx Tech CSC studies were based at UMass Chan and involved prospective sample collections with the goal of collecting data from diverse populations and settings. In order to accomplish this goal, the group included academic collaborators from UMass Chan, University of Massachusetts - Lowell (UML), Johns Hopkins University (JHU) and Northwestern University (NW) along with four Practice-Based Research Networks in Oregon, Iowa, Kansas and Georgia, all managing testing locations. Contracts were also established with University of California San Francisco (UCSF)/Eureka Digital Platform, Quest Diagnostics, Care Evolution Healthcare Technology, and the device manufacturers.

The UMass Chan team developed a platform study design to facilitate a series of device studies. Each study included key outcomes such as accuracy, speed, ease of use, accessibility and user acceptability. The master protocol and model consent form were then amended for each new device to be tested. Devices tested included a range of samples to be collected (e.g., nasal swabs or saliva) and collection methods (e.g., by the participant or a health care provider). All studies included a standard comparator assay and results were reported to participants.

In collaboration with Eureka the CSC developed a digital, paperless enrollment platform to allow for the consent process, data entry and data collection. The UMass Chan Institutional Review Board (IRB) served as the single IRB (sIRB) of record for all but a few studies that were reviewed by a commercial IRB. The UMass Chan sIRB reviewed 23 reliance agreements, 165 protocol submissions and amendments for a total of 240 final study documents.

As of May 2022, more than 15000 participants were enrolled to complete six studies on four devices, all of which received Emergency Use Authorizations from the Food and Drug Administration. Additionally, a multi-site biorepository was created that includes more than 1700 blood, mid-turbinate, saliva and nasopharyngeal samples for future research.

In order to coordinate this time sensitive national effort across academic medical centers, local provider networks, and vendors, the CSC established five teams: Study Design and Analysis (SDA), Study Management (SM), Study Logistics (SL), Community Health, Equity, and Engagement (CHEE) and Ethics and Human Subjects Oversight (EHSO). This manuscript will focus on the EHSO Team and describe one of a number of cases where input from the EHSO was sought.

## II. Ethics and Human Subjects Oversight (EHSO) Team

The EHSO provided ethics and regulatory expertise to any of the effort's stakeholders (faculty, staff, sponsors, vendors, collaborators). The RADx Team approach is distinct from an embedded ethics approach where an individual ethicist is embedded in the research team to integrate ethical considerations into the design and conduct of a study or research effort [4] [5]. The EHSO team had nearly 20 members from across the CSC and RADx Tech program, including faculty and staff and key collaborators (from UMass Chan, UMass Lowell, NIH, Emory, UCSF/Eureka) with expertise in research ethics, the review and oversight of human subjects research, adult and pediatric care, and study design. The EHSO brought key study stakeholders to the table with those with ethics and regulatory expertise. The EHSO also included a liaison to the CHEE team (JFH) given the overlapping interests and shared responsibilities.

EHSO met weekly during the same hour every week. Issues for discussion were brought to the group in advance or during the meeting. As studies launched and closed, four members of the team (the authors) moved to a drop-in format so CSC and others could drop in with “just-in-time” concerns. In addition to the weekly EHSO meetings some team members also attended a weekly CSC meeting meant to share progress and facilitate coordination across all teams and study leadership.

An early task of EHSO was to develop principles to guide the entire effort. See Box I.

Another early task was to work with the Study Management and Study Design teams on the development of the master protocol and consent documents. The goal was to create a master protocol and consent that could be efficiently amended by the study team and reviewed by the UMass Chan sIRB as each new collaborator and device joined the RADx Tech effort. The EHSO was asked to review key sections of the master protocol. Key ethical questions that led to discussion included: 1) when collecting more than one sample which sample should be tested with the novel device and in what order, 2) what number of samples is feasible to collect from each participant, and 3) if samples will be banked should it be a part of the master protocol or a separate protocol. The EHSO also reviewed content and format of the consent form and other participant facing materials. These documents were then shared with the CHEE team to assure that the documents met health literacy standards, were appropriate for a diverse audience and translated into additional languages as needed by the population to be approached for enrollment.

**Box I:** Guiding Principles for the Conduct of COVID-19 Test Us Research Studies.

This document is intended for all individuals involved in the design and execution of evaluations of novel point-of-care technologies under RADx Tech. The principles below should inform all decision points.

• RADx Tech's commitment to the rapid execution of much needed diagnostic testing for COVID-19 is balanced against our commitment and responsibility to protect research participants’ welfare, including voluntariness, privacy, protection of confidentiality, and safety.
• Sample populations and research participants should represent national diversity, including but not limited to age, race, ethnicity, sex, socioeconomic status, and local context.
• Research is to be conducted in collaboration with community, State, and Tribal stakeholders (as applicable) in a manner that is respectful and transparent.

• Respect for research participants begins at recruitment and extends through the dissemination of key study findings back to the community.
• Research should be offered to participants at times and locations that are convenient for them, which may include early mornings, evenings, weekends, and access via public transportation.
• The informed consent process is an active, ongoing process between the potential research participants and the study team. It includes a discussion about the RADx Tech effort, COVID-19, the specific test being studied, and reasons why they may or may not want to participate.
  ○ Study teams will work with potential participants to enhance their understanding of important aspects of the study in advance of enrollment.
  ○ Participant-facing materials should be in everyday language and use visual aids.
  ○ Participants should be informed up front that the goal of the research is to see if an experimental test works and if it is easy to use.
  ○ Participants should be informed about the performance characteristics of the experimental test and standard test to assess risks of participation.
• The approach to biobanking specimens for future use should be made clear to participants.

## III. Case Example: Disclosure of Research Results

The SDA team brought a key issue related to ethics and study design to the EHSO: return of research results. In mid-2020 there were few COVID-19 diagnostics available and those that were available and had emergency use authorization (EUA) were suboptimal in terms of their specificity and sensitivity [4]. The goal of the entire RADx Tech effort was to rapidly develop better diagnostics to address just this issue.

A key component of diagnostic test development is to determine how accurate the test is in identifying true positives. The next step is to assess performance in populations known to have the virus of interest and those who don't; the goal is to have a test that is both specific and sensitive [7]. An early ethical question in the development process was whether to share test results with asymptomatic individuals enrolling in RADx Tech trials. Each trial was designed to compare the result of the novel diagnostic with the current standard; with the possibility that the novel diagnostic would perform better than the standard. Under usual circumstances before providing a result back to the subject, that result would be confirmed by the “gold standard” (e.g., a test that has been cleared by the FDA and accepted as such). However, due to the novelty of COVID-19, all devices were under the Emergency Use Authorization (EUA) and none were FDA cleared, therefore one of the best available and widely used tests constituted the standard for confirming a positive result.

It was relatively quickly decided that concordant results would be returned to participants as soon as possible. The results being as accurate as possible under the circumstances. Delivering this information was a way to respect the subject's interest in knowing their infection status. Those who were found to be positive on both tests were educated about the implications of the result and referred to their primary care providers. It was not a difficult decision to agree that recommending an individual with two positive results isolate themselves to prevent infection to others.

Prior to the development of vaccines, getting a positive result meant a higher risk of developing severe disease leading to hospitalization and possibly death [8] [9]. At the beginning of the pandemic, a positive result may also cause high levels of stress and fear as there was significant higher morbidity and mortality associated with a positive COVID-19 diagnosis [10]. In addition to health consequences, a positive COVID-19 test had additional far reaching personal and professional implications including the recommendation to isolate for up to 10 days [11]. There were also social, family and economic repercussions of a positive result for individuals who lived with their elderly parents, were unable to isolate, and/or were care givers for individuals with comorbidities that increased the morbidity of the disease [12] [13] [14] [15].

The next challenge was what to do with discordant results, when the novel and standard test results were not the same. The team wanted to minimize the number of false positive results shared with study participants to balance the benefit of reducing community spread of the virus with the logistical, economic, caregiving and psychological burdens of isolation, the challenges of separating caregivers from elderly parents, and the possibility of losing income or even their jobs

Balancing the accuracy of the results with the burden of uncertainty for the participant, the EHSO recommended that when the standard test is positive but the novel test negative, the positive results should be shared with the participant, and they should be counseled to consider their results as positive for COVID-19. The EHSO also endorsed the idea of notifying the participant of the discordant results ASAP. Rather than delay the disclosure of *all* participant results to account for the extra time to conduct the tie breaker, EHSO recommended that the participants with test results that indicated the need for a tiebreaker to be notified of their discordant result and told what to expect next. The tie breaker protocol was described in the master consent form. A script was developed to notify them of the result, that a tie breaker would be conducted, and they would be alerted as soon as the results were available (See Box II).

**Box II:** Script for Conversations with Participants about Discordant Results.

*Test needs to be confirmed*

*As you may remember, you had two (2) COVID-19 tests done. One was the STANDARD test, and the other was the RESEARCH test. In your case, the STANDARD COVID-19 test was negative. However, the RESEARCH COVID-19 test was positive. If you hadn't been in this study, you would have only had the STANDARD COVID-19 test done, and we don't have enough experience with the RESEARCH COVID-19 test to know what that positive result means. For that reason, you can assume that you had a negative COVID-19 test result. To be cautious, we will be re-testing your original sample again with a different STANDARD COVID-19 test. We will contact you as soon as the result is available in about 2-3 days.*

*If you are concerned about the results of the RESEARCH COVID-19 test and would like to have another STANDARD COVID-19 test with a new sample, you can return to [research site] and receive a second COVID-19 STANDARD test at no cost.*

*We encourage you to wash your hands often, cough and/or sneeze into your elbow, wear a mask in public, and continue social distancing all per CDC guidelines website* https://www.cdc.gov/coronavirus/2019-ncov/prevent-getting-sick/prevention.html*.*

*Monitor your symptoms, and if you begin to feel unwell, please contact your primary care provider. If you do not have a primary care provider or health insurance, you can call [insert local provider contact] to establish care.*

*Seek immediate medical attention in an Emergency Department if you develop any severe symptoms, including but not limited to: difficulty breathing, chest pain, unable to eat or drink enough, or severe vomiting, diarrhea, or weakness. If possible, please call ahead prior to going to the Emergency Department.*

Participants were also told they could return to the study site for additional testing and/or be referred to other testing locations (e.g., local health department). Another consideration was the staff time needed to make these calls. The assumption was that the number of discordant results of this type were expected to be low and would not create an overwhelming burden on front line study staff to engage with each participant in this situation

Outcome: This approach was adopted, approved, and implemented. Less than 1% of the test results were discordant.

## IV. Discussion

Investigators faced ethical questions during the design and implementation stages of their human subjects research. The urgency to quickly obtain actionable guidance is amplified in the context of a public health crisis. The RADx Tech COVID-19 Clinical Studies Core sought input on a weekly basis for more than a year with early questions focused on development of subject-facing materials, return of research results, and assent processes when enrolling minors.

Having access to a pool of experts with ethical and regulatory knowledge who met weekly to tackle issues of importance to the investigators was critical to the overall success of the project. Investigators were able to efficiently make decisions, prepare IRB submissions, and obtain IRB approval. Over time EHSO developed a core group of members who were consistently in attendance each week, and when possible, questions were sent to the entire group ahead of a scheduled meeting to facilitate discussion. The shift across many domains to a virtual meeting space extended to EHSO enabling contributors from across the country.

EHSO discussions were robust and collaborative with input from all in attendance. Joining the varied expertise in research ethics, regulatory knowledge, adult and pediatric care, community engagement, and study design created an opportunity to provide focused and effective feedback from a variety of key perspectives to investigators. Investigators ultimately remained responsible for final decisions and IRB submissions.

## V. Conclusion

The overall RADx Tech effort led to six studies on four SARS-CoV-2 devices, all of which received EUA, plus a biorepository of nasal, saliva, and blood samples from more than 1700 participants. The RADx Tech effort was facilitated by the efficient use of a platform study protocol adapted to each trial, an efficient use of the sIRB model and the availability of the EHSO.

We believe the model of embedded ethics and regulatory expertise adopted by the RADx Tech effort was a resounding success and facilitated the rapid pace required during a public health emergency. This model should be considered for future national efforts as well as multi-site trials aimed at public health emergencies. Sponsors and institutions should consider supporting these efforts and/or encouraging investigators to connect with their local research ethics expertise.
